# Real-world treatment patterns and effectiveness of palbociclib plus an aromatase inhibitor in patients with metastatic breast cancer aged 75 years or older

**DOI:** 10.3389/fonc.2023.1237751

**Published:** 2023-09-28

**Authors:** Adam Brufsky, Xianchen Liu, Benjamin Li, Lynn McRoy, Connie Chen, Rachel M. Layman, Hope S. Rugo

**Affiliations:** ^1^ Division of Hematology/Oncology, Department of Medicine, UPMC Hillman Cancer Center, University of Pittsburgh Medical Center, Pittsburgh, PA, United States; ^2^ Department of Oncology Medical Affairs, Pfizer Inc., New York, NY, United States; ^3^ Department of Breast Medical Oncology, The University of Texas MD Anderson Cancer Center, Houston, TX, United States; ^4^ Division of Hematology/Oncology, Department of Medicine, University of California San Francisco Helen Diller Family Comprehensive Cancer Center, San Francisco, CA, United States

**Keywords:** palbociclib, elderly, real-world, metastatic breast cancer, HR+/HER2−

## Abstract

**Background:**

Elderly patients are generally underrepresented in oncology clinical trials; therefore, real-world data are needed to inform clinical management of elderly patients with hormone receptor–positive/human epidermal growth factor receptor 2–negative (HR+/HER2−) metastatic breast cancer (mBC). This subanalysis of the P-REALITY X study (NCT05361655) evaluated palbociclib treatment patterns and comparative effectiveness of palbociclib plus an aromatase inhibitor (AI) versus an AI alone among patients with HR+/HER2− mBC aged ≥ 75 years treated in routine clinical practice in the United States.

**Methods:**

This retrospective observational cohort study used electronic health records from the Flatiron Health Analytic Database. Palbociclib treatment patterns, overall survival (OS), real-world progression-free survival (rwPFS), and time to chemotherapy (TTC) were evaluated. Three methods were used for comparative analyses: (1) an unadjusted analysis, (2) stabilized inverse probability treatment weighting (sIPTW; primary analysis), and (3) propensity score matching (PSM; sensitivity analysis).

**Results:**

A total of 961 patients aged ≥ 75 years with HR+/HER2− mBC were identified who started palbociclib plus an AI (n = 313) or an AI alone (n = 648) as first-line (1L) therapy between February 2015 and March 2020 (data cut-off: September 30, 2020). Among patients in the palbociclib plus an AI group with a documented palbociclib starting dose (n = 306), approximately 75% started palbociclib at 125 mg/day, and approximately 40% experienced dose adjustment. After sIPTW, patients treated with palbociclib plus an AI versus an AI alone had significantly improved OS (median of 43.0 vs. 32.4 months; hazard ratio [HR], 0.66 [95% confidence interval (CI), 0.51–0.84]; *P* = 0.0007), rwPFS (median of 20.0 vs. 15.0 months; HR, 0.72 (0.59–0.89); *P* = 0.0021), and TTC (median of 40.2 vs. 27.4 months; HR, 0.69 [0.55–0.87]; *P* = 0.0014). These significant improvements in OS, rwPFS, and TTC remained consistent in the unadjusted analysis and after PSM.

**Conclusion:**

This real-world comparative analysis demonstrated that 1L palbociclib plus an AI is associated with improved effectiveness compared with an AI alone among patients with HR+/HER2− mBC aged ≥ 75 years. These findings support palbociclib plus an AI as a standard-of-care 1L treatment for elderly patients with HR+/HER2− mBC.

## 1 Introduction

Breast cancer is the leading cancer diagnosis in the United States (US) and commonly affects older adults, with a median age of 63 years at diagnosis (1, 2). Patients aged ≥ 65 years accounted for approximately 45% of new breast cancer diagnoses (65–74 years, 26.5%; ≥ 75 years, 18.9%) and 62% of breast cancer deaths (65–74 years, 24.4%; ≥ 75 years, 38.0%) in the US in recent years (1). Hormone receptor-positive/human epidermal growth factor receptor 2-negative (HR+/HER2−) disease is the most common breast cancer subtype, and the proportion of patients with the HR+/HER2− subtype increases with age, from 64.8% among patients aged < 50 years to 80.1% among patients aged ≥ 75 years (3). Despite the high incidence of breast and other cancers in the elderly population (1, 4), these patients have been largely underrepresented in clinical trials in oncology, including registrational trials for new cancer therapies (5–7). For example, an analysis of accrual to breast cancer trials conducted by the Alliance for Clinical Trials in Oncology found that < 20% of trial participants were ≥ 65 years of age and < 10% were ≥ 70 years of age (8). Thus, a greater understanding of treatment benefits and risks is needed for the elderly population of patients with breast cancer.

The combination of a cyclin-dependent kinase 4/6 (CDK4/6) inhibitor and an aromatase inhibitor (AI) is a standard first-line (1L) therapy for patients with HR+/HER2− metastatic breast cancer (mBC) (9). Palbociclib, a first-in-class CDK4/6 inhibitor, is approved in the US for the treatment of adult patients with HR+/HER2− advanced or mBC in combination with an AI as the initial endocrine-based regimen or with fulvestrant in patients with disease progression after prior endocrine therapy (ET) (10, 11). The 1L indication for palbociclib plus an AI is supported by results from the phase 3 PALOMA-2 trial (10), which demonstrated significant improvement in progression-free survival (PFS) in patients with estrogen receptor-positive/HER2− advanced breast cancer receiving palbociclib plus letrozole compared with those receiving placebo plus letrozole (27.6 vs. 14.5 months; hazard ratio [HR], 0.563 [95% confidence interval (CI), 0.461−0.687]; *P* < 0.0001) (12). In the PALOMA-2 trial, median overall survival (OS) was numerically longer in the palbociclib plus letrozole group compared with the placebo plus letrozole group, although the difference was not statistically significant (53.9 vs. 51.2 months; *P* > 0.05) (13). In the subgroup of patients aged ≥ 65 years in the PALOMA-2 trial, median PFS was significantly prolonged with palbociclib plus letrozole (30.6 vs. 19.1 months; HR, 0.60 [95% CI, 0.43−0.86]; *P* < 0.005) (12), and median OS showed numerical improvement (58.6 vs. 47.4 months; HR, 0.871 [0.624−1.216]) (13).

Clinical trials often have stringent eligibility criteria that can limit the diversity in demographic and clinical characteristics of enrolled patient populations (14–16). As a result, clinical trial findings can have limited generalizability to real-world clinical practice (14, 17). Therefore, real-world evidence is needed to inform the use of therapies in patient populations that are often underrepresented in clinical trials, such as older adults (18, 19). Palbociclib REAl-world first-LIne comparaTive effectiveness studY eXtended (P-REALITY X) used the Flatiron Database to compare the effectiveness of 1L palbociclib plus an AI versus an AI alone in patients with HR+/HER2− mBC in routine clinical practice in the US (20). Notably, in the P-REALITY X study, the median age of patients after stabilized inverse probability treatment weighting (sIPTW) was 70 years in both treatment groups, which is 8–9 years older than the median age in the PALOMA-2 trial (20, 21). After sIPTW, median OS was significantly prolonged in patients treated with palbociclib plus an AI versus an AI alone (49.1 vs. 43.2 months; HR, 0.76 [95% CI, 0.65–0.87]; *P* < 0.0001) (20). Patients treated with palbociclib plus an AI also had significantly prolonged median real-world PFS (rwPFS) after sIPTW than those treated with an AI alone (19.3 vs. 13.9 months; HR, 0.70 [95% CI, 0.62–0.78]; *P* < 0.0001).

Prior real-world studies have examined the comparative effectiveness of 1L palbociclib plus ET versus ET alone in elderly patients with HR+/HER2− mBC (22, 23). For example, a recent retrospective analysis of the Flatiron Database found that women aged ≥ 65 years with HR+/HER2− mBC treated with 1L palbociclib plus letrozole had significantly prolonged median rwPFS (22.2 vs. 15.8 months; HR, 0.59 [95% CI, 0.47–0.74]; *P* < 0.001) and median OS (not reached [NR] vs. 43.4 months; HR, 0.55 [0.42–0.72]; *P* < 0.001) after sIPTW compared with those treated with letrozole alone (23). In addition, a retrospective analysis of the Survey Epidemiology and End Results (SEER)-Medicare database demonstrated a 41% lower rate of mortality in women aged ≥ 65 years with HR+/HER2− mBC receiving 1L treatment with a CDK4/6 inhibitor plus ET versus ET alone using multivariable Cox regression analysis (adjusted HR, 0.590 [95% CI, 0.423–0.823]) (22); notably, palbociclib accounted for approximately 90% of CDK4/6 inhibitor use in this study (22, 24).

Treating elderly patients with mBC presents many challenges, including frequent comorbidities, increased risk of drug-induced toxicity, and concerns regarding polypharmacy and drug-drug interactions (25, 26). Therefore, more robust long-term data from large real-world studies are needed to better understand dosing patterns and clinical outcomes of elderly patients with HR+/HER2− mBC receiving 1L palbociclib plus an AI in routine clinical practice, especially among those aged ≥ 75 years. The analysis presented herein aimed to describe palbociclib dose patterns and compare the effectiveness of 1L palbociclib plus an AI versus an AI alone in the subgroup of patients aged ≥ 75 years in the P-REALITY X study.

## 2 Methods

### 2.1 Study design and data source

P-REALITY X (NCT05361655) was a retrospective observational cohort study of electronic health records (EHRs) obtained from the Flatiron Health Analytic Database. This longitudinal database contains de-identified patient data from > 280 cancer clinics representing > 3 million actively treated patients with cancer in the US. Detailed methods for the P-REALITY X study have been published previously (20). In the subanalysis of P-REALITY X presented herein, we identified patients aged ≥ 75 years with HR+/HER2− mBC who started palbociclib plus an AI or an AI alone as 1L therapy between February 2015 and March 2020. Patients were evaluated from the start of treatment with palbociclib plus an AI or an AI alone to September 30, 2020 (data cut-off date), death, or last visit, whichever came first.

### 2.2 Outcomes

Outcomes evaluated in this analysis included palbociclib treatment patterns, OS, rwPFS, and time to chemotherapy (TTC). Palbociclib treatment patterns, including the starting dose and dose adjustments, were captured from EHRs during the observation period. OS was defined as the number of months from the start of treatment with palbociclib plus an AI or an AI alone until death. The date of death was determined using a composite of multiple data sources, which were benchmarked against the National Death Index. Patients who did not die were censored at the data cut-off date. rwPFS was defined as the number of months from the start of palbociclib plus an AI or an AI alone to death due to any cause or disease progression, whichever occurred first (20, 27). Disease progression was assessed by the treating clinician based on radiology, tissue biopsy, laboratory evidence, or clinical assessment. If patients did not die or experience disease progression, those with ≥ 2 lines of therapy (LoT) were censored at the date of initiation of the next LoT, and those with 1 LoT were censored at their last visit date during the study period. TTC was defined as the number of months from the start of palbociclib plus an AI or an AI alone to chemotherapy, death from any cause, last visit, or end of the study, whichever occurred first. If a patient did not have evidence of subsequent chemotherapy and did not die, the patient was censored at the latest available date or data cut-off date, whichever occurred later. Notably, safety was not assessed in this analysis because safety data were not available in the database for the P-REALITY X study.

### 2.3 Statistical analysis

Descriptive statistics were used to describe patient characteristics and palbociclib treatment patterns. Three methods were used for comparative analyses between treatment groups: (1) an unadjusted analysis that did not control for baseline demographic and clinical characteristics, (2) sIPTW (primary analysis) to balance baseline demographic and clinical characteristics, and (3) 1:1 propensity score matching (PSM) as a sensitivity analysis. Both sIPTW and PSM methodologies used propensity scores, defined as the probability of treatment assignment based on observed baseline demographic and clinical variables (28, 29). Propensity scores were computed using a multivariable binomial logistic regression model, which included the following variables: age group, sex, race/ethnicity, practice type, disease stage at initial diagnosis, Eastern Cooperative Oncology Group performance status, bone disease, visceral disease, the interval from initial breast cancer diagnosis to mBC diagnosis, and the number of metastatic sites. Time-to-event endpoints, including OS, rwPFS, and TTC, were summarized using the weighted Kaplan–Meier method and displayed graphically. The weighted Cox proportional hazards model was used to compute HR and corresponding 95% CI for time-to-event endpoints.

## 3 Results

### 3.1 Patients

A total of 961 patients aged ≥ 75 years with HR+/HER2− mBC were included in this analysis, of whom 313 (32.6%) received palbociclib plus an AI and 648 (67.4%) received an AI alone as 1L therapy (**Table 1**). The median age was 80.0 years for both groups and > 90% of patients were treated in the community practice setting. Median follow-up duration before sIPTW or PSM adjustment was 23.7 months and 21.4 months for patients treated with palbociclib plus an AI and an AI alone, respectively. More patients treated with palbociclib plus an AI (n = 133/313; 42.5%) had *de novo* mBC compared with those treated with an AI alone (n = 219/648; 33.8%) before sIPTW and PSM analysis. Patient characteristics were generally balanced between treatment groups after sIPTW and PSM (**Table 1**).

**Table 1:** Patient characteristics.

Characteristic	Unadjusted total cohort			Cohort after sIPTW			Cohort after PSM		
	Palbociclib + AI (n = 313)	AI alone (n = 648)	Standardized difference	Palbociclib + AI (n = 371)	AI alone (n = 287)	Standardized difference	Palbociclib + AI (n = 252)	AI alone (n = 252)	Standardized difference
Age at mBC diagnosis, years^a^ Mean (SD) Median (IQR)	79.4 (2.9) 80.0 (5.0)	80.2 (2.5) 80.0 (3.0)	-0.2738	79.5 (3.2) 80.0 (5.0)	80.2 (1.6) 80.0 (3.0)	-0.2475	79.5 (2.9) 80.0 (5.0)	80.3 (2.4) 80.0 (3.0)	-0.3031
Female sex,^a^ n (%)	309 (98.7)	643 (99.2)	0.0503	367 (98.9)	284 (98.9)	0.0032	250 (99.2)	251 (99.6)	0.0516
Race,^a^ n (%) White Black Other	214 (68.4) 19 (6.1) 80 (25.6)	454 (70.1) 48 (7.4) 146 (22.5)	-0.0366 -0.0534 0.0709	254 (68.5) 27 (7.3) 90 (24.2)	199 (69.4) 20 (7.0) 68 (23.6)	-0.0197 0.0092 0.0159	178 (70.6) 14 (5.6) 60 (23.8)	180 (71.4) 11 (4.4) 61 (24.2)	-0.0175 0.0549 -0.0093
Practice type,^a^ n (%) Community Academic	294 (93.9) 19 (6.1)	617 (95.2) 31 (4.8)	-0.0568	352 (94.8) 19 (5.2)	271 (94.6) 16 (5.4)	0.0118	240 (95.2) 12 (4.8)	240 (95.2) 12 (4.8)	0.0000
Insurance Commercial health plan plus any other Commercial health plan Medicare Medicaid Other payer type	112 (35.8) 57 (18.2) 17 (5.4) 2 (0.6) 125 (39.9)	229 (35.3) 108 (16.7) 43 (6.6) 2 (0.3) 266 (41.0)	0.0093 0.0407 -0.0506 0.0481 -0.0227	129 (34.8) 72 (19.5) 20 (5.3) 3 (0.7) 147 (39.7)	100 (34.9) 50 (17.6) 19 (6.5) 1 (0.3) 117 (40.8)	-0.0017 0.0483 -0.0499 0.0612 -0.0213	88 (34.9) 44 (17.5) 13 (5.2) 2 (0.8) 105 (41.7)	83 (32.9) 45 (17.9) 15 (6.0) 0 109 (43.3)	0.0419 -0.0104 -0.0347 0.1265 -0.0321
Disease stage at initial diagnosis,^a^ n (%) I II III IV Not documented	40 (12.8) 78 (24.9) 26 (8.3) 133 (42.5) 36 (11.5)	93 (14.4) 152 (23.5) 87 (13.4) 219 (33.8) 97 (15.0)	-0.0459 0.0342 -0.1650 0.1797 -0.1025	56 (15.1) 84 (22.7) 46 (12.4) 127 (34.2) 58 (15.6)	41 (14.2) 68 (23.6) 34 (11.8) 104 (36.1) 41 (14.2)	0.0246 -0.0229 0.0166 -0.0401 0.0413	38 (15.1) 65 (25.8) 21 (8.3) 96 (38.1) 32 (12.7)	39 (15.5) 66 (26.2) 20 (7.9) 96 (38.1) 31 (12.3)	-0.0110 -0.0090 0.0145 0.0000 0.0120
ECOG PS,^a^ n (%) 0 1 2, 3, or 4 Not documented	98 (31.3) 87 (27.8) 53 (16.9) 75 (24.0)	127 (19.6) 136 (21.0) 160 (24.7) 225 (34.7)	0.2713 0.1590 -0.1920 -0.2380	83 (22.5) 87 (23.3) 89 (23.9) 113 (30.4)	67 (23.2) 67 (23.4) 64 (22.2) 89 (31.2)	-0.0186 -0.0012 0.0393 -0.0178	73 (29.0) 64 (25.4) 48 (19.0) 67 (26.6)	75 (29.8) 66 (26.2) 43 (17.1) 68 (27.0)	-0.0174 -0.0181 0.0516 -0.0090
Visceral disease,^a,b^ n (%)	106 (33.9)	170 (26.2)	-0.1670	109 (29.4)	84 (29.2)	-0.0047	82 (32.5)	84 (33.3)	0.0169
Bone-only metastasis,^a,c^ n (%)	123 (39.3)	253 (39.0)	-0.0052	137 (36.8)	111 (38.6)	0.0380	103 (40.9)	101 (40.1)	-0.0162
Brain metastases, n (%)	4 (1.3)	12 (1.9)	0.0463	5 (1.3)	6 (2.0)	0.0599	3 (1.2)	5 (2.0)	0.0635
Disease-free interval,^a^ n (%) *De novo* mBC ≤ 1 year > 1–5 years > 5 years Not documented	133 (42.5) 5 (1.6) 37 (11.8) 138 (44.1) 0	219 (33.8) 26 (4.0) 139 (21.5) 261 (40.3) 3 (0.5)	0.1797 -0.1467 -0.2608 0.0772 -0.0964	127 (34.2) 12 (3.2) 55 (14.9) 177 (47.8) 0	104 (36.1) 10 (3.6) 57 (20.0) 114 (39.8) 1 (0.4)	-0.0401 -0.0243 -0.1370 0.1601 -0.0875	96 (38.1) 5 (2.0) 33 (13.1) 118 (46.8) 0	96 (38.1) 8 (3.2) 48 (19.0) 100 (39.7) 0	0.0000 -0.0752 -0.1626 0.1446 N/A
NCI comorbidity index, mean (SD)	0.4 (0.6)	0.5 (0.6)	-0.2003	0.5 (0.7)	0.5 (0.4)	-0.0432	0.4 (0.6)	0.5 (0.6)	-0.1071
Number of metastatic sites,^a,d^ n (%) 1 2 3 4 ≥ 5 Not documented	150 (47.9) 85 (27.2) 43 (13.7) 8 (2.6) 11 (3.5) 16 (5.1)	357 (55.1) 129 (19.9) 53 (8.2) 13 (2.0) 8 (1.2) 88 (13.6)	-0.1438 0.1715 0.1787 0.0368 0.1502 -0.2941	187 (50.4) 84 (22.7) 36 (9.6) 7 (2.0) 12 (3.1) 45 (12.2)	150 (52.2) 64 (22.4) 30 (10.5) 7 (2.4) 5 (1.6) 31 (10.9)	-0.0345 0.0077 -0.0299 -0.0325 0.0978 0.0407	128 (50.8) 65 (25.8) 31 (12.3) 5 (2.0) 8 (3.2) 15 (6.0)	125 (49.6) 63 (25.0) 30 (11.9) 8 (3.2) 7 (2.8) 19 (7.5)	0.0238 0.0182 0.0122 -0.0752 0.0234 -0.0633
Median follow-up duration (IQR), months	23.7 (23.4)	21.4 (27.1)	NA	22.6 (24.0)	21.4 (27.0)	NA	23.5 (23.7)	22.4 (27.6)	NA

a Variable used in propensity score estimation.

b Visceral disease was defined as metastatic disease in the lung and/or liver; patients could have other sites of metastases. No visceral disease was defined as no lung or liver metastases.

c Bone-only disease was defined as metastatic disease in the bone only.

d Multiple metastases at the same site were counted as 1 site (e.g., if a patient had 3 bone metastases in the spine, it was considered only 1 site).

AI, aromatase inhibitor; ECOG PS, Eastern Cooperative Oncology Group Performance Status; IQR, interquartile range; mBC, metastatic breast cancer; NA, not applicable; NCI, National Cancer Institute; PSM, propensity score matching; SD, standard deviation; sIPTW, stabilized inverse probability treatment weighting.

### 3.2 Palbociclib starting dose and dose adjustment

Among the 306 patients treated with palbociclib plus an AI who had a documented palbociclib starting dose, 230 (75.2%) patients started palbociclib at 125 mg/day, 53 (17.3%) at 100 mg/day, and 23 (7.5%) at 75 mg/day (**Table 2**). Patient characteristics by initial palbociclib dose are presented in **Supplementary Table S1**. There was some variation in patient characteristics across dose cohorts, such as differences in median age and the proportions of patients with visceral or bone-only disease. However, the small sample sizes of patients with a starting dose of 100 or 75 mg/day precluded us from comparative analyses.

**Table 2:** Palbociclib dose adjustments.

Dose modification	Initial dose		
	125 mg/day (n = 230)	100 mg/day (n = 53)	75 mg/day (n = 23)
**Any dose change, n (%)**	97 (42.2)	19 (35.8)	5 (21.7)
125 to 100 mg/day only	54 (23.5)	—	—
125 to 100 to 75 mg/day only	25 (10.9)	—	—
125 to 75 mg/day only	10 (4.3)	—	—
100 to 75 mg/day only	—	15 (28.3)	—
100 to 125 mg/day only	—	2 (3.8)	—
75 to 100 mg/day only	—	—	2 (8.7)
75 to 100 to 125 mg/day only	—	—	1 (4.3)
Other change	8 (3.5)	2 (3.8)	2 (8.7)
**Dose change direction, n (%)**			
Adjustment^a^	8 (3.5)	2 (3.8)	2 (8.7)
Increase	0	2 (3.8)	3 (13.0)
Reduction	89 (38.7)	15 (28.3)	0
No change	133 (57.8)	34 (64.2)	18 (78.3)
**Median (IQR) number of days to the first dose adjustment among patients with any dose change**	72.0 (101.0)	59.0 (163.0)	62.0 (135.0)
**Number of dose adjustments (among all patients)**			
Median	0	0	0
Mean (SD)	0.61 (0.89)	0.38 (0.53)	0.43 (0.99)
Range	0–6	0–2	0–4

a Refers to cases where dose change directions included an unknown change or a combination of dose reductions and increases over time (e.g., dose reduction followed by a dose increase, or vice versa).

IQR, interquartile range; SD, standard deviation.

**Figure 1:**
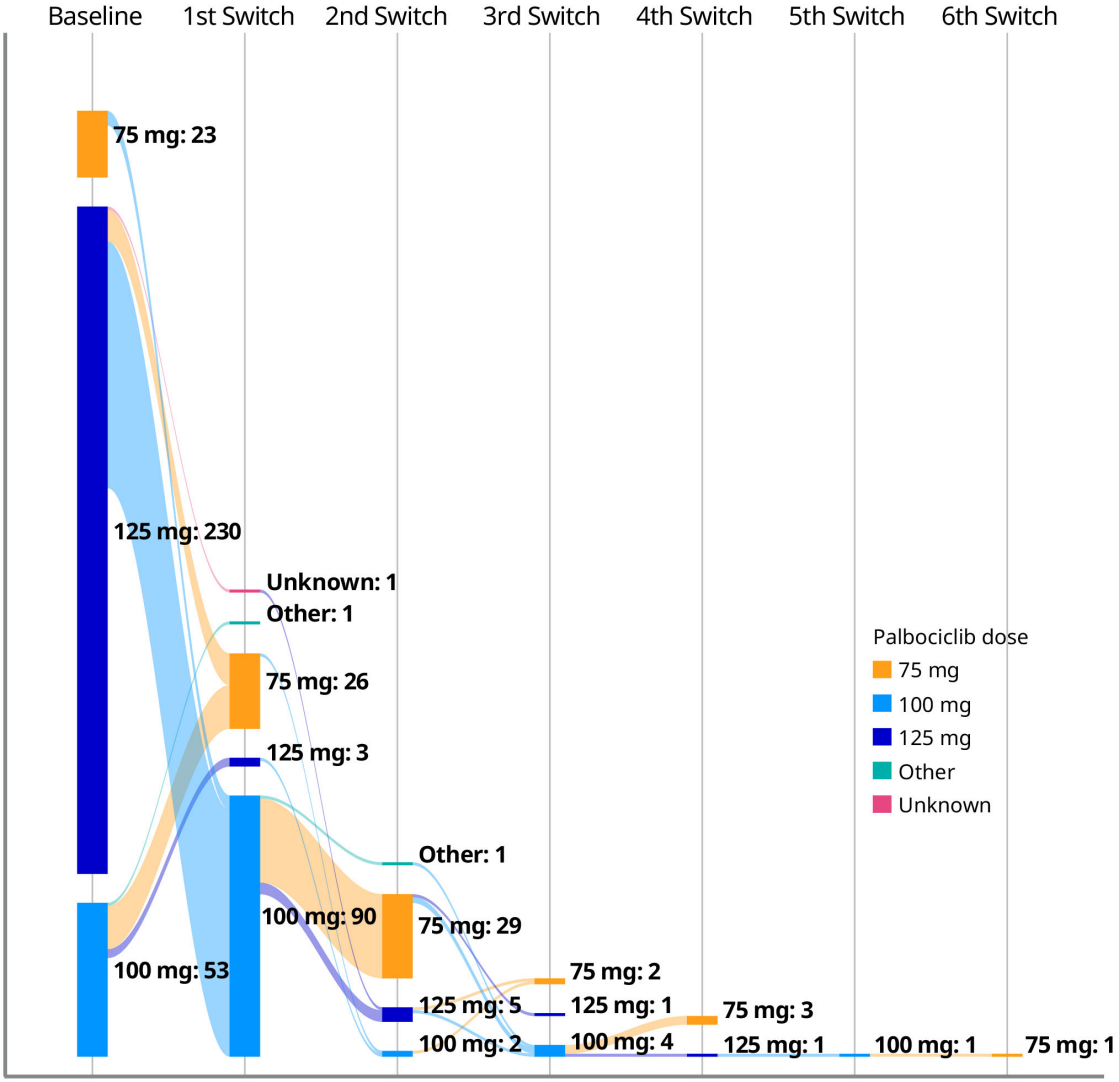
Palbociclib dose adjustments among patients with a documented palbociclib starting dose (n = 306).

In total, 121 of 306 patients (39.5%) with a documented palbociclib starting dose experienced dose adjustments. Of the patients who initiated palbociclib at a dose of 125, 100, and 75 mg/day, 97 (42.2%), 19 (35.8%), and 5 (21.7%) patients experienced dose adjustments, respectively (**Figure 1**). For patients who received an initial palbociclib dose of 125, 100, and 75 mg/day and experienced any dose adjustment, the median number of days to the first dose adjustment was 72, 59, and 62 days, respectively.

### 3.3 Overall survival

**Figure 2:**
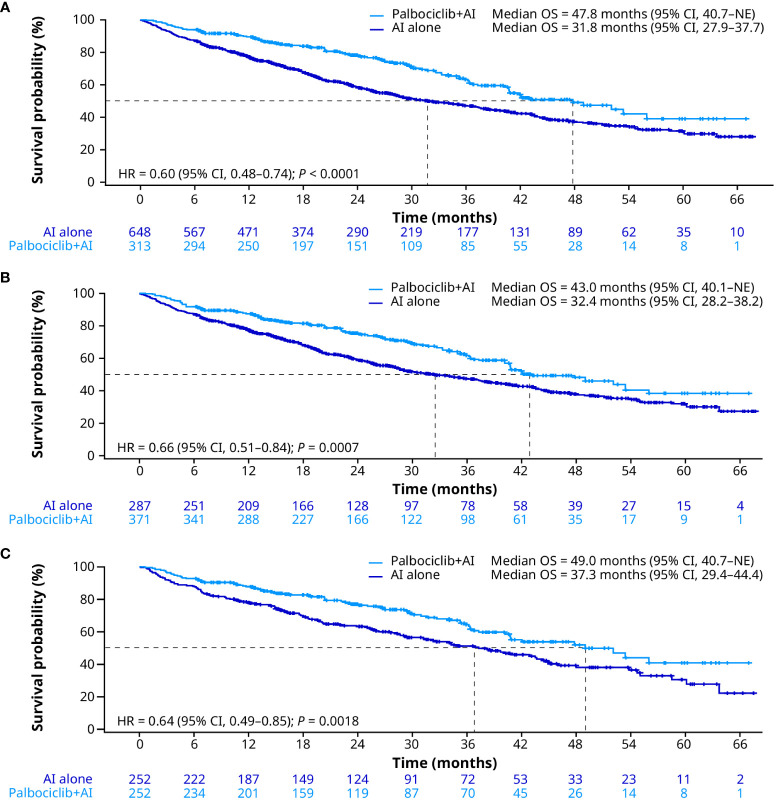
Overall survival in the unadjusted **(A)**, sIPTW **(B)**, and PSM **(C)** analyses. AI, aromatase inhibitor; CI, confidence interval; HR, hazard ratio; OS, overall survival; PSM, propensity score matching; sIPTW, stabilized inverse probability treatment weighting.

In the unadjusted analysis, median OS was significantly prolonged for patients treated with palbociclib plus an AI versus an AI alone (47.8 months [95% CI, 40.7–not estimable (NE)] vs. 31.8 months [27.9–37.7]; HR, 0.60 [0.48–0.74]; *P* < 0.0001; **Figure 2A**). After sIPTW, median OS was 43.0 months (95% CI, 40.1–NE) in the palbociclib plus an AI group and 32.4 months (28.2–38.2) in the AI group (HR, 0.66 [0.51–0.84]; *P* = 0.0007; **Figure 2B**). After PSM, median OS was 49.0 months (95% CI, 40.7–NE) in the palbociclib plus an AI group versus 37.3 months (29.4–44.4) in the AI group (HR, 0.64 [0.49–0.85]; *P* = 0.0018; **Figure 2C**).

### 3.4 Real-world progression-free survival

**Figure 3:**
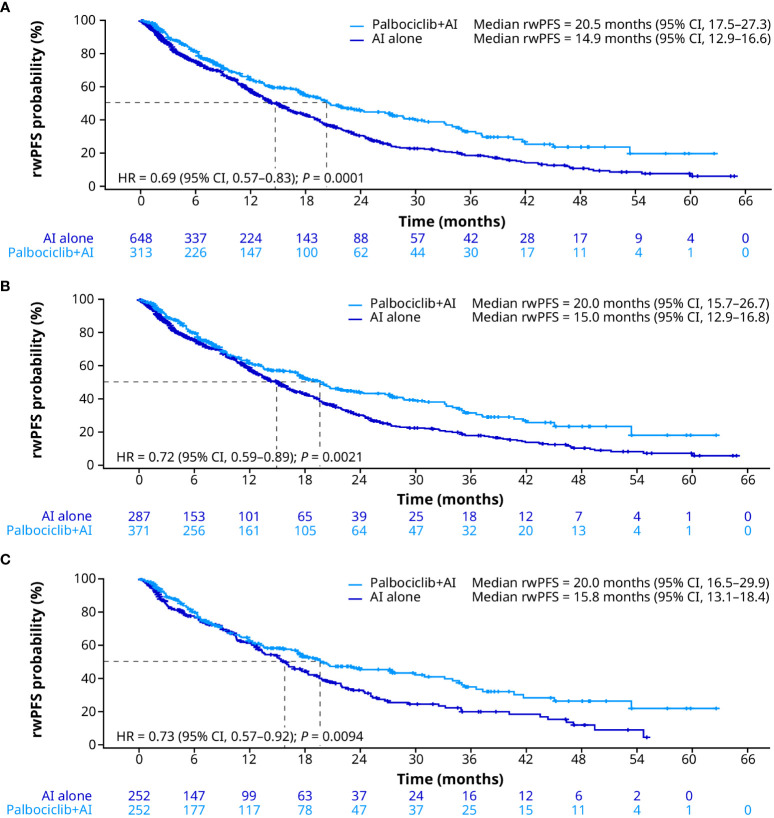
Real-world progression-free survival in the unadjusted **(A)**, sIPTW **(B)**, and PSM **(C)** analyses. AI, aromatase inhibitor; CI, confidence interval; HR, hazard ratio; PSM, propensity score matching; rwPFS, real-world progression-free survival; sIPTW, stabilized inverse probability treatment weighting.

In the unadjusted analysis, patients treated with palbociclib plus an AI had significantly longer median rwPFS than patients treated with an AI alone (20.5 months [95% CI, 17.5–27.3] vs. 14.9 months [12.9–16.6]; HR, 0.69 [0.57–0.83]; *P* = 0.0001; **Figure 3A**). After sIPTW, median rwPFS was 20.0 months (95% CI, 15.7–26.7) and 15.0 months (12.9–16.8) in the palbociclib plus an AI group and the AI group, respectively (HR, 0.72 [0.59–0.89]; *P* = 0.0021; **Figure 3B**). After PSM, median rwPFS was 20.0 months (95% CI, 16.5–29.9) in patients treated with palbociclib plus an AI and 15.8 months (13.1–18.4) in patients treated with an AI alone (HR, 0.73 [0.57–0.92]; *P* = 0.0094; **Figure 3C**).

### 3.5 Subsequent treatments

During the follow-up period, 136 of 313 (43.5%) patients in the palbociclib plus an AI group and 361 of 648 (55.7%) patients in the AI alone group received subsequent treatment. Second-line (2L) treatments following 1L palbociclib plus an AI or an AI alone are presented in **Table 3**. Among patients in the palbociclib plus an AI group receiving any 2L treatment (n = 136), 44.1% received a CDK4/6 inhibitor and 19.1% received chemotherapy as 2L treatment. Among patients in the AI alone group receiving any 2L treatment (n = 361), 39.6% received a CDK4/6 inhibitor and 10.0% received chemotherapy as 2L treatment.

**Table 3:** Subsequent second-line anticancer treatments.

Treatments	Unadjusted total cohort	
	Palbociclib + AI (n = 313)	AI alone (n = 648)
1L treatment only,^a^ n (%)	177 (56.5)	297 (45.8)
Any 2L treatment received,^b^ n (%)	136 (43.5)	361 (55.7)
CDK4/6 inhibitor	60/136 (44.1)	143/361 (39.6)
Chemotherapy	26/136 (19.1)	36/361 (10.0)
Endocrine therapy alone	37/136 (27.2)	174/361 (48.2)
Other anticancer treatments	22/136 (16.2)	31/361 (8.6)

a Includes patients who continued treatment, died, or were censored in the 1L setting.

b Patients could have received > 1 category of 2L treatment.

1L, first-line; 2L, second-line; AI, aromatase inhibitor; CDK4/6, cyclin-dependent kinase 4/6.

### 3.6 Time to chemotherapy

**Figure 4:**
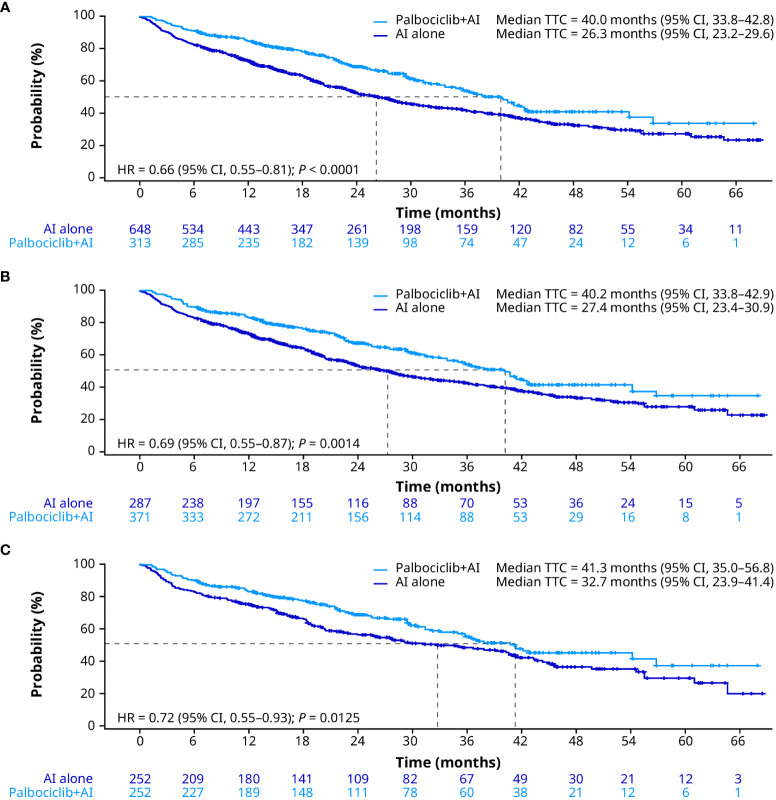
Time to chemotherapy in the unadjusted **(A)**, sIPTW **(B)**, and PSM **(C)** analyses. AI, aromatase inhibitor; CI, confidence interval; HR, hazard ratio; PSM, propensity score matching; sIPTW, stabilized inverse probability treatment weighting; TTC, time to chemotherapy.

Consistent with OS and rwPFS, median TTC was significantly prolonged for patients treated with palbociclib plus an AI compared with patients treated with an AI alone in the unadjusted analysis (40.0 months [95% CI, 33.8–42.8] vs. 26.3 months [23.2–29.6]; HR, 0.66 [0.55–0.81]; *P* < 0.0001; **Figure 4A**). After sIPTW, median TTC was 40.2 months (95% CI, 33.8–42.9) in patients treated with palbociclib plus an AI and 27.4 months (23.4–30.9) in patients treated with an AI alone (HR, 0.69 [0.55–0.87]; *P* = 0.0014; **Figure 4B**). After PSM, median TTC was 41.3 months (95% CI, 35.0–56.8) in the palbociclib plus an AI group and 32.7 months (23.9–41.4) in the AI group (HR, 0.72 [0.55–0.93]; *P* = 0.0125; **Figure 4C**). In addition, of patients treated with an AI alone, 13.6% of patients received palbociclib combination therapy prior to chemotherapy.

## 4 Discussion

Treatment decision-making for elderly patients with HR+/HER2− mBC requires particularly careful consideration of many factors, including comorbidities, possible drug-drug interactions, functional status, and the likelihood of drug-induced toxicities (25, 26). Unfortunately, data to inform the clinical management of elderly patients are limited because these patients are generally underrepresented in clinical trials in oncology (5–7). As a result, real-world data are needed to help evaluate the effectiveness and inform on the use of palbociclib in elderly patients with HR+/HER2− mBC. In this analysis, we evaluated real-world palbociclib treatment patterns and compared the effectiveness of 1L palbociclib plus an AI versus an AI alone in patients aged ≥ 75 years with HR+/HER2− mBC in the P-REALITY X study. We found that approximately 75% of patients aged ≥ 75 years with HR+/HER2− mBC started palbociclib at a dose of 125 mg/day in the real-world setting, and approximately 40% of patients experienced dose adjustment. Compared with an AI alone, 1L palbociclib plus an AI was associated with significantly improved OS, rwPFS, and TTC before and after sIPTW or PSM adjustment. Palbociclib plus an AI is indicated as initial endocrine-based therapy for the treatment of adult patients with HR+/HER2− mBC, irrespective of patient age (10), and these real-world data further support the use of this regimen as a standard 1L treatment option for the elderly population.

The PFS data from this real-world analysis are generally consistent with the results from other clinical trials and real-world studies that compared 1L palbociclib plus AI versus AI alone in elderly patients with HR+/HER2− mBC (12, 23, 27, 30). In the subgroup of patients aged ≥ 65 years in the PALOMA-2 study (n = 262), PFS was significantly prolonged in patients receiving 1L palbociclib plus letrozole versus placebo plus letrozole, at a median of 30.6 months versus 19.1 months, respectively (HR, 0.60 [95% CI, 0.43−0.86]; *P* < 0.005) (12). In addition, a pooled analysis of data from the PALOMA-1 and PALOMA-2 studies showed significant improvement in median PFS with palbociclib plus letrozole versus placebo plus letrozole among patients aged 65−74 years (n = 256; 27.5 vs. 21.8 months; HR, 0.66 [95% CI, 0.45−0.97]; *P* = 0.016) and ≥ 75 years (n = 82; NR vs. 10.9 months; HR, 0.31 [0.16−0.61]; *P* < 0.001) (30). Furthermore, previous retrospective analyses of the Flatiron Database comparing the effectiveness of 1L palbociclib plus letrozole versus letrozole alone demonstrated a benefit with palbociclib in sIPTW-adjusted rwPFS among patients with HR+/HER2− mBC who were aged ≥ 65 years (median of 22.2 vs. 15.8 months; HR, 0.59 [95% CI, 0.47–0.74]; *P* < 0.001) (23) or ≥ 70 years (HR, 0.58 [0.46−0.74]) (27). Thus, PFS data from clinical trials, previous real-world studies, and the real-world analysis presented herein collectively support using palbociclib in combination with an AI as a 1L treatment for elderly patients with HR+/HER2– mBC.

In this study, median OS after sIPTW (primary analysis) was significantly improved with palbociclib plus an AI versus an AI alone. A similar OS benefit was observed after sIPTW in prior retrospective analyses of the Flatiron Database that compared the effectiveness of 1L palbociclib plus letrozole versus letrozole alone in patients with HR+/HER2− mBC who were aged ≥ 65 years (NR vs. 43.4 months; HR, 0.55 [95% CI, 0.42–0.72]; *P* < 0.001) (23) or ≥ 70 years (HR, 0.55 [0.40−0.77]) (27). Moreover, a SEER-Medicare population-based study found that 1L treatment with a CDK4/6 inhibitor (predominantly palbociclib) plus ET versus ET alone was associated with a significant OS benefit (adjusted HR, 0.590 [95% CI, 0.423–0.823]) in women aged ≥ 65 years with HR+/HER2− mBC (22). A similar trend in OS was observed in the PALOMA-2 trial, which showed numerical, albeit not significant, improvement with palbociclib plus letrozole versus placebo plus letrozole in the subgroup of patients aged ≥ 65 years (median of 58.6 vs. 47.4 months; HR, 0.871 [95% CI, 0.624−1.216]) (13).

In addition to the comparative studies described above, several prior single-arm, real-world studies have evaluated rwPFS and OS in elderly patients with advanced or mBC receiving palbociclib (31–35). In a national United Kingdom retrospective study of patients aged ≥ 75 years with ER+/HER2− advanced breast cancer receiving 1L palbociclib plus AI (N = 276), 12- and 24-month rwPFS rates were 75.9% and 64.9%, respectively, and OS rates were 85.1% and 74.0%, respectively (31). A retrospective analysis at a French comprehensive cancer center evaluated outcomes in patients aged ≥ 70 years who received palbociclib plus ET for HR+/HER2− advanced breast cancer (32). In this heavily pretreated cohort (N = 52), with a median of 3 (range, 0−9) previous treatments for advanced metastatic disease, median PFS was 9 months (95% CI, 6−NR), and median OS was NR (22 months−NE). In a retrospective analysis of patients receiving palbociclib in any LoT at MD Anderson Cancer Center (N = 605), older patients (using an age cut-off of either 65 or 70 years) had significantly improved rwPFS compared with younger patients; however, the multivariable analysis did not find age to be significantly associated with disease progression (33). An analysis using real-world data retrieved from the Dutch Institute for Clinical Auditing medicines program for patients with advanced breast cancer receiving palbociclib (N = 598) found the median OS of patients aged ≥ 70 years to be 20.7 months, which was not significantly different from that observed in patients aged < 70 years (26.7 months; *P* = 0.051) (34). Similarly, a retrospective review of a multicenter institutional database evaluating outcomes with palbociclib plus ET in patients with HR+/HER2− advanced breast cancer (N = 271) found no significant differences between patients aged ≥ 65 versus < 65 years in rwPFS (8 vs. 10 months) or OS (22 vs. 34 months; *P* = 0.221) (35). Taken together, these real-world data shed further insight into the effectiveness of palbociclib in routine clinical practice in the elderly population and suggest that elderly patients may derive a similar benefit from palbociclib as younger patients.

Toxicity management is particularly important when treating elderly patients with HR+/HER2− mBC (25, 26). For example, postponing the initiation of salvage chemotherapy can help spare patients from the toxicities and detrimental effects on quality of life associated with chemotherapy (25, 36). In our study, TTC was significantly prolonged with palbociclib plus an AI versus an AI alone before and after sIPTW and PSM adjustment. We also analyzed palbociclib dose reductions, which can be used to mitigate hematologic adverse events associated with palbociclib treatment, such as neutropenia (37). In prior real-world studies evaluating palbociclib plus ET use in elderly patients, palbociclib dose reductions were most frequently attributed to neutropenia, but could also result from other hematologic or non-hematologic toxicities, such as thrombocytopenia or fatigue (31, 35). We found that approximately 25% of patients had a starting palbociclib dose lower than 125 mg/day, and approximately 39% of patients who started with a dose of 125 mg/day experienced a dose reduction. In the palbociclib plus letrozole arm in the PALOMA-2 trial, a similar proportion of patients (39.4%) experienced a dose reduction (12). Importantly, dose reductions did not compromise efficacy in PALOMA-2, and PFS was similar among patients who did or did not experience a dose reduction (37). In a retrospective analysis of the MD Anderson Cancer Center database, palbociclib dose reductions were more commonly observed in elderly versus younger patients. However, these dose reductions did not significantly affect rwPFS (adjusted HR, 0.7; *P* = 0.07) (33). Further studies are needed to explore the effect of palbociclib dose reductions on other effectiveness and safety outcomes in elderly patients. Detailed safety assessments were not possible in our analysis because safety data were not retrieved/abstracted in the database for the P-REALITY X study. Although beyond the scope of our current study, several studies in the real-world or clinical setting have previously demonstrated that palbociclib is generally well-tolerated in elderly patients (30–33, 35, 38) and that quality of life and functional status are preserved in elderly patients receiving treatment with palbociclib (30, 39). Prior analyses of clinical trial or real-world data have reported rates of treatment discontinuation due to toxicity ranging from 3% to 13% among elderly patients receiving palbociclib plus ET (30–32, 35).

To our knowledge, the P-REALITY X study is the largest multisite comparative effectiveness study to date comparing 1L palbociclib plus an AI versus an AI alone for patients with HR+/HER2− mBC in a real-world setting. Strengths of the present study include the diversity of the patient population captured in the Flatiron Database, the large sample size of patients aged ≥ 75 years (n = 961), the contemporaneous control group, and the long median follow-up time. The OS endpoint in the Flatiron Database is a consensus variable across multiple data sources (including the Social Security Death Index, obituaries, EHRs, and commercial death data) and validated through comparisons with the National Death Index (40, 41). Furthermore, the consistency in significant findings in the unadjusted analysis, the primary analysis with sIPTW, and the sensitivity analysis with PSM contribute to the study’s internal validity. However, this real-world study also has several potential limitations. This was a retrospective database analysis, which may have potential bias in treatment selection, incomplete or missing data, limited information on comorbidities, and potential for inaccurate data capture. Disease progression was not assessed as scheduled in clinical trials and was not based on Response Evaluation Criteria in Solid Tumors; therefore, rwPFS data are limited by each treating clinician’s interpretation of radiographic scans or pathology results and the lack of standardization in the timing of these assessments. Although sIPTW and PSM were used to balance patient characteristics, the potential effects of unmeasured confounders could not be adjusted for in the analysis. Lastly, results from this analysis may not be generalizable to patients outside the Flatiron network.

## 5 Conclusions

Overall, this comparative analysis of 1L palbociclib plus an AI versus an AI alone indicates that palbociclib plus an AI is associated with improved effectiveness with prolonged OS, rwPFS, and TTC in patients with HR+/HER2− mBC who are aged ≥ 75 years. These findings support palbociclib in combination with endocrine therapy as a standard-of-care treatment for elderly patients with HR+/HER2− mBC.

## Data availability statement

The data that support the findings of this study have been originated by Flatiron Health, Inc. These de-identified data may be made available upon request, and are subject to a license agreement with Flatiron Health; interested researchers should contact <DataAccess@flatiron.com> to determine licensing terms and get the training, data dictionary, validation, and data sets. The Flatiron Health Analytic Database can be contacted at https://flatiron.com/contact/.

## Author contributions

All authors listed have made a substantial, direct, and intellectual contribution to the work, and approved it for publication.

## Supplementary material

Supplementary Table 1 Patient characteristics by initial palbociclib dose.
