# Supplementary material for: Real-world treatment patterns and effectiveness of palbociclib plus an aromatase inhibitor in patients with metastatic breast cancer aged 75 years or older
Source: Front Oncol. 2023 Sep 28;13:1237751. doi: 10.3389/fonc.2023.1237751 (PMC10569486; doi:10.3389/fonc.2023.1237751)
Supplement: Supplementary Table 1 — Patient characteristics by initial palbociclib dose. [file Table_1.docx]

**SUPPLEMENTARY TABLE S1 Patient characteristics by initial palbociclib dose.**

|  | **Initial dose** | | | **Overall**  **(n = 306)** |
| --- | --- | --- | --- | --- |
|  | **125 mg/day**  **(n = 230)** | **100 mg/day**  **(n = 53)** | **75 mg/day**  **(n = 23)** |  |
| Age at mBC diagnosis, years  Mean (SD)  Median (IQR) | 79.1 (2.8)  79.0 (4.0) | 79.6 (2.8)  80.0 (5.0) | 81.5 (2.7)  82.0 (4.0) | 79.4 (2.8)  80.0 (5.0) |
| Female sex, n (%) | 226 (98.3) | 53 (100.0) | 23 (100.0) | 302 (98.7) |
| Race, n (%)  White  Black  Other | 163 (70.9)  15 (6.5)  52 (22.6) | 31 (58.5)  1 (1.9)  21 (39.6) | 16 (69.6)  2 (8.7)  5 (21.7) | 210 (68.6)  18 (5.9)  78 (25.5) |
| Practice type, n (%)  Community  Academic | 215 (93.5)  15 (6.5) | 50 (94.3)  3 (5.7) | 23 (100)  0 | 288 (94.1)  18 (5.9) |
| Insurance  Commercial health plan plus any other  Commercial health plan  Medicare  Medicaid  Other payer type | 85 (37.0)  37 (16.1)  13 (5.7)  2 (0.9)  93 (40.4) | 16 (30.2)  11 (20.8)  3 (5.7)  0  23 (43.4) | 7 (30.4)  7 (30.4)  1 (4.3)  0  8 (34.8) | 108 (35.3)  55 (18.0)  17 (5.6)  2 (0.7)  124 (40.5) |
| Disease stage at initial diagnosis, n (%)  I  II  III  IV  Not documented | 30 (13.0)  58 (25.2)  16 (7.0)  99 (43.0)  27 (11.7) | 6 (11.3)  13 (24.5)  6 (11.3)  20 (37.7)  8 (15.1) | 4 (17.4)  5 (21.7)  3 (13.0)  11 (47.8)  0 | 40 (13.1)  76 (24.8)  25 (8.2)  130 (42.5)  35 (11.4) |
| ECOG PS, n (%)  0  1  2, 3, or 4  Not documented | 78 (33.9)  61 (26.5)  38 (16.5)  53 (23.0) | 12 (22.6)  18 (34.0)  8 (15.1)  15 (28.3) | 7 (30.4)  6 (26.1)  6 (26.1)  4 (17.4) | 97 (31.7)  85 (27.8)  52 (17.0)  72 (23.5) |
| Visceral disease,^a^ n (%) | 70 (30.4) | 24 (45.3) | 10 (43.5) | 104 (34.0) |
| Bone-only metastasis,^b^ n (%) | 96 (41.7) | 15 (28.3) | 9 (39.1) | 120 (39.2) |
| Brain metastases, n (%) | 2 (0.9) | 2 (3.8) | 0 | 4 (1.3) |
| Disease-free interval, n (%)  De novo mBC  ≤ 1 year  > 1–5 years  > 5 years  Not documented | 99 (43.0)  4 (1.7)  26 (11.3)  101 (43.9)  0 | 20 (37.7)  1 (1.9)  7 (13.2)  25 (47.2)  0 | 11 (47.8)  0  3 (13.0)  9 (39.1)  0 | 130 (42.5)  5 (1.6)  36 (11.8)  135 (44.1)  0 |
| NCI comorbidity index, mean (SD) | 0.4 (0.6) | 0.4 (0.5) | 0.5 (0.7) | 0.4 (0.6) |
| Number of metastatic sites,^c^ n (%)  1  2  3  4  ≥ 5  Not documented | 117 (50.9)  63 (27.4)  28 (12.2)  6 (2.6)  6 (2.6)  10 (4.3) | 19 (35.8)  13 (24.5)  13 (24.5)  1 (1.9)  4 (7.5)  3 (5.7) | 10 (43.5)  8 (34.8)  2 (8.7)  1 (4.3)  1 (4.3)  1 (4.3) | 146 (47.7)  84 (27.5)  43 (14.1)  8 (2.6)  11 (3.6)  14 (4.6) |
| Median follow-up duration (IQR), months | 23.8 (23.5) | 23.7 (24.5) | 24.2 (30.6) | 23.8 (24.0) |

^a^Visceral disease was defined as metastatic disease in the lung and/or liver; patients could have other sites of metastases. No visceral disease was defined as no lung or liver metastases.

^b^Bone-only disease was defined as metastatic disease in the bone only.

^c^Multiple metastases at the same site were counted as 1 site (e.g., if a patient had 3 bone metastases in the spine, it was considered only 1 site).

AI, aromatase inhibitor; ECOG PS, Eastern Cooperative Oncology Group Performance Status; IQR, interquartile range; mBC, metastatic breast cancer; NCI, National Cancer Institute; SD, standard deviation.
